# Response to letter to Editor by Gatterer and Burtscher concerning splenic contraction in different situations

**DOI:** 10.1007/s00421-021-04704-6

**Published:** 2021-06-04

**Authors:** Erika Schagatay, Pontus Holmström

**Affiliations:** Department of Health Sciences, Mid Sweden Universty, House D, Studentplan 4, 831 25 Östersund, Sweden

Dear Editor,

We read with interest the letter to editor by Hannes Gatterer, and Martin Burtscher, as a response to our recent paper (Lodin-Sundström et al. 2021). They bring up the important point that our data do not entirely allow to clearly distinguish between the exercise- and the hypoxia-induced effects on splenic contraction and/or its interaction. We agree that our study does not fully determine, merely suggests, that exercise in hypoxia is different from exercise in normoxia, concerning its effects on the spleen. As suggested, to accurately conclude if the high altitude simulation or the exercise itself is the main stimulus—or the two in combination—follow-up studies are required, using each of these separately and in combination.

Nevertheless, our aim was not to compare splenic responses between exercise and hypoxia to elucidate a presumed additive or synergistic effect. Instead, our aim was to reveal whether there is an increased effect of the two different stimuli when they occur together, or an all or nothing response. Our design would resemble the situation as it occurs naturally at high altitude, wherein exercise is superimposed on the already existing hypobaric hypoxia, as individuals (e.g., trekkers and climbers) transition between rest in hypoxia and exercise in hypoxia. The stepwise response when superimposing and removing exercise to a hypoxic stimulus shows that the spleen response is graded and likely able to fine-tune the amount of circulating red cells to meet the oxygen demands in the short term. This effect has been suggested by several of our previous field studies (Richardson et al. 2007; Schagatay et al. 2020; Holmström et al. 2021a, b). However, in those studies, the hypoxic stimulus was chronic and it could not be ruled out that other changes affected baseline hemoglobin concentration (Hb; e.g., elevations due to dehydration or induced by enhanced erythropoiesis) and possibly spleen size.

There are numerous ways of combining stimuli for elucidating the control mechanisms of spleen contraction and it would be challenging to accomplish it all in one study unless an extremely long protocol is used, a situation in which there would likely be unwanted influence of exhaustion. Nonetheless, we are currently working on research questions pertaining to distinguishing the effects of single and combined stimuli of different levels. Our preliminary results show that in elite athletes, who are more familiar with maximal effort exercise than maximal apnea, normoxic intense exercise clearly induced a stronger spleen contraction accompanied by a stronger Hb elevation than the hypoxia induced by maximal apnea at rest. Moreover, other preliminary results show that the spleen contraction is stepwise more pronounced with increasing work intensity, which is in line with the findings on two different exercise intensities in the study by Stewart et al. (2003). This is supporting the conclusion by Gatterer and Burtscher on an exercise intensity-dependent response, when comparing studies by Lodin-Sundström et al. (2021) with those by Stewart et al. (2003). We want to emphasize, however, that interpretations based on comparisons between studies of absolute spleen volumes in response to various stimuli should be done with caution, as the individual differences in both spleen volume and subsequent Hb elevations are large. For solid conclusions on mechanisms, participants need to act as their own controls, or very large samples have to be used.

The aim of our recent paper was not to determine the splenic contraction resulting from different levels of exercise, but the response to two different stimuli (Lodin-Sundström et al. 2021). Concerning the combined effects of high altitude hypoxia with other stimuli, we found in a previous study where we examined exercise at different altitudes, that resting spleen volume was reduced with incremental ascent and spleen volume was further reduced with exercise at all altitudes, while Hb values changed in the opposite direction (Schagatay et al. 2020). In another study, wherein apnea was implemented to induce spleen contraction at different altitudes (Holmström et al. 2021a; b), we observed a similar stepwise more-pronounced tonic spleen contraction with incremental ascent, but also here another stimulus—apnea—in general added to the contraction, attesting to a tonic splenic contraction of about − 14%/1000 m of ascent. The phenomenon was in fact noted already in 2007 in a study only measuring Hb (Richardson et al. 2007). The current study (Lodin-Sundström et al. 2021) shows that also in the short term, with progressively smaller baseline spleen volume, the spleen contraction resulting from apnea is also reduced, suggesting that a smaller capacity for contraction remains when more of the red cell reservoir is recruited during resting conditions due to hypoxia per-se.

We agree that more research is warranted to identify all potential mechanisms leading to spleen contraction at high altitude and in other situations. A study involving paired samples during exercise of the same absolute and relative work rate at different (real or simulated) altitudes in randomized order still remains to be conducted. We hope that the novel and interesting insights in this field will lead to more studies being conducted by different groups. A structured meta-analysis would be useful, when more material is available on this topic. There are in fact several important questions left to be answered concerning spleen contraction and its effects—a relatively newly discovered phenomenon compared to most physiological defense mechanisms—including the issue of to what extent it is genetically determined or an effect of training, while there is certainly some influence of both. Similarly, it is important to determine its magnitude and beneficial effects, if any, for the endurance athlete.

Erika Schagatay and Pontus Holmström, on behalf of all authors.
